# Ultrasound-Assisted Extraction of Bioactive Compounds from Annatto Seeds, Evaluation of Their Antimicrobial and Antioxidant Activity, and Identification of Main Compounds by LC/ESI-MS Analysis

**DOI:** 10.1155/2019/3721828

**Published:** 2019-07-16

**Authors:** Julian Quintero Quiroz, Ana Maria Naranjo Duran, Mariluz Silva Garcia, Gelmy Luz Ciro Gomez, John Jairo Rojas Camargo

**Affiliations:** ^1^College of Pharmaceutical and Food Sciences, University of Antioquia, Street 67, No. 53-108, Medellin, Colombia; ^2^Institute of Food Science and Technology (INTAL), Carrera 50g No. 12S-91, Itagüi, Colombia

## Abstract

This study evaluated the antimicrobial activity (i.e., against* Bacillus cereus* and* Staphylococcus aureus*) and the antioxidant activity (i.e., ABTS, FRAP, and DPPH) of annatto seeds extract obtained by ultrasound-assisted extraction. A response surface design with three levels such as pH (2-11), solvent concentration (50-96 %), seed-to-solvent ratio (1:2–1:10), and treatment time (0-30 min) was employed to determine the optimal experimental conditions. Thus, a pH of 7.0, seed-to-solvent ratio of 1:7, and treatment time of 20 min were selected as optimal rendering an extract having a 0.62% of bixin, 3.81 mg gallic acid/mg equivalent of polyphenol compounds (ABTS 1035.7, FRAP 424.7, and DPPH 1161.5 *μ*M trolox/L), and a minimal inhibitory concentration against* Bacillus cereus* and* Staphylococcus aureus* of 32 and 16 mg/L, respectively. Further, the main bioactive compounds identified by LC/ESI-MS were bixin and catechin, chlorogenic acid, chrysin, butein, hypolaetin, licochalcone A, and xanthohumol.

## 1. Introduction

The seeds of annatto (*Bixa orellana L.*) are widely employed as a dye for foods such as butter, cheese, soft drinks, and confectionery. The pigment obtained from this plant is rich in carotenoids (i.e., bixin) and polyphenol compounds such as hypolatin and caffeic acid [1]. The antioxidant ability of the extract is attributed to the high conjugation of these compounds and their ability to quench singlet oxygen, deactivation of the excited triplet state of sensitizers usually associated with photosensitization, sweeping free radicals, and denaturation of proteins from cell membranes. Further, the extract exhibits anti-inflammatory, antioxidant, and antimicrobial activities [2]. However, the extraction method affects the functional properties of the extract. Traditionally, different extraction methods for bioactive compounds include percolation, solvent extraction or leaching, enzyme-assisted extraction, and liquid-liquid microextraction [3]. However, conventional methods, such as solvent extraction or leaching, require a long processing time, exposing the bioactive compounds to degradation factors leading to a low efficiency [4].

To date, ultrasound and microwaves-assisted extractions have been reported for the extraction of several components. Particularly, mechanical methods, spouted bed, ultrasound-assisted extraction, microwave-assisted extraction, and supercritical fluids (CO_2_) have been employed for annatto seed extraction [3, 5–7]. Nonetheless, these studies have been focused exclusively on the extraction of the pigment, leaving aside the evaluation of the effect of the extraction treatment on activities such as antimicrobial and antioxidant. The increase of the extraction yield of the bioactive compounds obtained by the ultrasound and microwave-assisted techniques is attributed to the acceleration of mass transfer from the solid-phase to the liquid-phase [3]. The ultrasound-assisted extraction (UAE) creates cavitation of small bubbles in the solvent due to the passage of ultrasound waves allowing for a greater penetration of the solvent within the material increasing the surface area. On the other hand, microwave-assisted extraction implies the formation of high-energy electromagnetic waves changing the molecular rotation and ionic mobility of the medium without destroying the sample, resulting in heating and possibly denaturation of bioactive compounds. The last two methods force the migration of all active compounds rapidly from the solid-phase to the solvent-phase. However, the UAE shows a better protection of those compounds due to the lower temperature generated in the medium [8, 9].

Further, not only the evaluation of the functional activity of the extracts, but also the identification of the compounds responsible for that activity is crucial. In general, bixin, phenolic acids, lignans, and flavonoids are the main bioactive compounds. However, the isolation and identification of these compounds require techniques having a high separation efficiency since coeluting compounds could be missed [10]. Currently, HPLC coupled with mass spectrometry at atmospheric pressure chemical ionization (APCI) or electrospray ionization (ESI) represents the most selective analytical technique for the identification and quantification of compounds isolated from plants and food sources. The identification of carotenoids and polyphenol compounds by LC–ESI-MS using a LTQ Orbitrap XL renders an extremely high mass accuracy (<5 ppm of resolution). This technique is capable of multiple levels of fragmentation to reveal the molecular weight (MW) of unknown compounds. Hence, by using this technique, unknown compounds are identified on the basis of nominal MW and MS/MS fragmentation [10].

The goal of this study was to enhance the extraction of bioactive compounds from the seeds of annatto and optimize the extraction conditions (i.e., solvent concentration, pH, treatment time, and seed-to-solvent ratio) as compared to a conventional method. Further, it is expected that the antimicrobial and antioxidant activity is due to the high yield of extracted compounds as identified by LC/ESI-MS.

## 2. Materials and Methods

### 2.1. Materials

Annatto seeds were purchased from a farmers market of Medellín (Colombia), which were sun-dried until reaching a moisture content of 10.58 ± 0.98 %. All the reagents used were synthesis grade.

### 2.2. Experimental Design

The UAE conditions were optimized using a response surface plot experimental design (Box-Behnken (BBD)), using the Design Expert Software® (Version 8.0.6, Stat-Ease, USA). The dependent variables such as treatment time (min) (X_1,_ 0-30), pH (X_2_, 4-11), solvent concentration (%) (X_3_: 50-96), and seed-to-solvent ratio (X_4_, 2-10) were studied. The solvent used was absolute ethanol (lot k4958171742, Merck Darmstadt, Germany). With the independent variables studied, the statistical program established 30 experimental units, which are presented in Table 1 with their respective randomization. The two independent variables studied were the bixin content (Bix) and the total polyphenol content found by the Folin-Ciocalteu method, using a uv/vis spectrophotometer (UV-1700, Shimadzu) [11].

The operational conditions of the equipment used for the UAE were a frequency of 37 KHz and a power of 320 W (Elmasonic E30h). Adjustment of the pH was done with 0.1 M acetic acid (lot k47109963539, Merck Darmstadt, Germany) and 0.1 M sodium hydroxide solution (lot b0895598319, Merck Darmstadt, Germany). The method of multiple regressions of least squares was used in order to optimize the extraction conditions and investigate the effect of the independent variables. The experimental data was adjusted by the second-order polynomial equation (see (1)) by comparison of the determination coefficient (r^2^) and the adjusted determination coefficient (r^2^-adj). The quadratic model follows the expression

(1) Y=β0+β1X1+β2X2+β3X3+β4X4+β11X11+β22X22+β33X32+β44X42+β12X1X2+β13X1X3+β14X1X4+β23X2X3+β24X2X4+β34X3X4

where Y represents the predicted response, *β*_0_ is the intercept of the model, *β*_1_, *β*_2_, *β*_3_, *β*_4_, *β*_11_, *β*_22_, *β*_33_, *β*_44_ y *β*_12_, *β*_13_, *β*_14_, *β*_23_, *β*_24_, and, *β*_34_ are the linear and interaction coefficients, respectively, and X_1_, X_2_, X_3_, and, X_4_ correspond to the independent variables. Analysis of variance (ANOVA) was used to analyze the significant effects (with a confidence level of 95%) of the independent variables over the dependent variables. Once the significant mathematical models were obtained, the extraction process was optimized, seeking to maximize the extraction of polyphenols and bixin (weight of 1 for both) and minimizing the seed-to-solvent ratio. The calculation of the relative and absolute errors was accomplished between the responses predicted by the model versus the ones obtained experimentally under optimal conditions.

### 2.3. Characterization of Optimized Annatto Seed Extracts

The antioxidant and antimicrobial activities of the optimized extracts obtained by the UAE were compared with (i) an extract obtained by conventional extraction (with ethanol, seeds-to-solvent ratio of 1:2, and continuous agitation for 48 h) and (ii) previous results from an optimized microwave-assisted extraction [12]. The results are presented as means and standard deviation (SD). The analysis was performed using the Statgraphics® Centurion XVI software.

### 2.4. Quantification of Total Polyphenol

The total phenolic concentration in the annatto seed extract was measured by the Folin-Ciocalteu method [13]. Twenty microliter of sample was mixed with in 1.58 *μ*L of distilled water, 100 *μ*L of Folin-Ciocalteu phenol reagent (lot hc43368401, Merck Darmstadt, Germany) and 300 *μ*L of 20 % sodium carbonate (lot a0594092339, Merck Darmstadt, Germany). The absorbance was read at 725 nm in a UV/VIS spectrophotometer (UV-1700, Shimadzu) after 1h of storage under darkness. The experiments were performed in triplicate and the results were expressed as mg of gallic acid (GA) (lot SLBM8746V, Merck Darmstadt, Germany) per gram of seeds (mgGA/g seed).

#### 2.4.1. Quantification of Bixin

100 *μ*L of sample with 2 mL of tetrahydrofuran (lot dg643, Merck Darmstadt, Germany) and 10 mL of acetone (lot k43912814243, Merck Darmstadt, Germany) were mixed to obtain an absorbance of less than 0.15 at 487 nm taken in a UV/VIS spectrophotometer (UV-1700, Shimadzu). The concentration of bixin in the sample was determined using the following equation [14]:

(2) $$\begin{array}{c} Bixin\;\;\left(\;mg/mL\;\right)=\frac{A*100*V}{A_{1cm}^{1\%}*100} \end{array}$$

where

*A*_1*cm*_^1%^ = 3090 (1g/100 mL)^−1^*∗*1cm ^−1^ (specific absorptivity coefficient of bixin in acetone) [14];

A = absorbance value of the sample;

V = dilution volume of the sample (mL).

#### 2.4.2. Antioxidant Activity, ABTS^+^ Method

The ABTS assay was performed following the method described by Contreras* et al*. 2011 [15]. One hundred microliter of sample (diluted appropriately with water) was mixed with 1 mL of ABTS^+^ solution, which was prepared with ABTS standard (2,2′-Azino-bis(3-ethylbenzothiazoline-6-sulfonic acid) diammonium salt, lot slbp9592v, sigma-Aldrich, St. Lois, USA) diluted in ethanol. The change in coloration was read after 30 min at 730 nm in a spectrophotometer (UV-1700, Shimadzu). The results were expressed as trolox equivalents (TE) (6-hydroxy-2,5,7,8-tetramethylchromane-2-carboxylic acid to 97%, lot stbb6668, sigma-Aldrich, St. Lois, USA) or TE *μ*mol /L.

#### 2.4.3. Antioxidant Activity, Ferric Reducing Antioxidant Power (FRAP) Method

The FRAP was measured according to Benzie and Strain (1996) with modifications [16]. Briefly, 30 *μ*L of sample, 90 *μ*L of deionized water (diluted appropriately with water), and 900 *μ*L of the FRAP reagent (prewarmed at 37°C) were mixed. The sample was incubated for 30 minutes at 37°C. Then, the change in coloration was read at 593 nm in a spectrophotometer (UV-1700, Shimadzu). A Trolox (6-hydroxy-2,5,7,8-tetramethylchromane-2-carboxylic acid to 97%, lot stbb6668, sigma-Aldrich, St. Lois, USA) calibration curve was obtained for quantification purposes and the results were expressed as TE *μ*mol /L.

#### 2.4.4. Antioxidant Activity, DPPH Method

The DPPH assay was performed as previously described [17]. Briefly, 2 mL of 0.5 mM 2,2-diphenyl-1-picrylhydrazyl or DPPH (067k lot 1154, sigma-Aldrich, St. Lois, USA) reagent was mixed with 2 mL of methanol (lot l823009611, Merck Darmstadt, Germany) and 0.2 mL of sample (diluted appropriately with water). The change in coloration was read after 30 min of incubation in darkness at 517 nm in a spectrophotometer (UV-1700, Shimadzu). A Trolox (6-hydroxy-2,5,7,8-tetramethylchromane-2-carboxylic acid to 97%, lot stbb6668, sigma-Aldrich, St. Lois, USA) calibration curve was obtained for quantification purposes and the results were expressed as TE *μ*mol/L.

#### 2.4.5. Minimal Inhibitory Concentration of Annatto Seed Extracts

The antibacterial activity was evaluated against* Bacillus cereus* (ATCC 11778) and* Staphylococcus aureus (*ATCC 6538). Bacterial strains were incubated for 24 h at 37°C in a Mueller-Hinton broth (lot b1223098542, Merck Darmstadt, Germany) and adjusted to a McFarland scale 0.5 (10^6^ CFU/mL). Samples were dissolved in dimethylsulfoxide (DMSO) (lot 190260, Merck Darmstadt, Germany) to reach concentrations ranging from 4 to 4096 mg/L. Ninety-six well microplates were prepared by adding 20 *μ*L of sample, 120 *μ*L of Mueller-Hinton broth, and 10 *μ*L of inoculum. The positive and negatives controls were prepared according to previous reports [12]. After incubation for 5 h at 37°C, 25 *μ*L of 3-(4,5-dimethylthiazol-2-yl)-2,5-diphenyl-tetrazolium bromide (MTT) (lot p31b064, Alfa Aesar, Tewksbury, USA) was added to each well and incubated for 1 h. The minimum inhibitory concentration (MIC) was considered as the concentration of the first well that did not suffer from any color change (from yellow to purple). The procedure was repeated three times for each microorganism and pH (4, 7, and 11) [2].

### 2.5. Identification of Bioactive Compounds (Polyphenols and Bixin) by LC/ESI-MS Analysis

The extract obtained at the optimal operational conditions by UAE was analyzed for the identification of the bioactive compounds. LC/ESI-MS analyses were performed using an UHPLC (Thermo Fisher Scientific Ultimate 3000) coupled with an orbitrap Q-exactive mass spectrometer (Thermo Fisher Scientific, Waltham, MA, USA). Chromatographic separation was done by revers-phase elution equipped with a SiliaChrom® Plus C18 (SILICYCLE) HPLC column (4.6 x 150 mm, 5 *μ*m, 100 Å). The mobile phase consisted of a combination of A (0.1% formic acid (lot 798381, PanReac AppliChem, Barcelona, Spain) in water) and B (0.1% formic acid in acetonitrile (lot 830491, PanReac AppliChem, Barcelona, Spain)) at a flow rate of 0.2 mL min^−1^ (injection volume of 90 *μ*L). The gradient was linear from 0% to 40% B for 60 min, followed by 100% B from 65 to 70 min, and held with 100% B from 70 to 75 min. The composition of the eluent was then restored to 100% A for 2 min, and then the system was reequilibrated for 14 min. The mass spectrometer was equipped with an electrospray interface (ESI), which was operated in positive-ion mode and controlled by the Xcalibur® software (version 2.0). The parameters of the ESI source were as follows: positive-ion mode spray voltage of 4.0 kV, capillary voltage of 49 V, capillary temperature of 275°C, and tube lens of 120 V. The identification of the polyphenol compounds was based on their molecular weight, using the Phenol-Explorer database containing 502 polyphenols. Bixin was identified according to its molecular weight (394.5 g/mol) [10, 18].

## 3. Results and Discussion

### 3.1. Experimental Design for Optimization of UAE

The experimental matrix was composed of 30 runs. The experiments were performed in triplicate and Table 1 lists the experimental conditions.

The ANOVA was used to evaluate the significance of the quadratic polynomial models. For each term in the models, a large F-value and a small* p*-value would imply a more significant effect on the respective response variable [19]. The ANOVA table for the UAE (Table 2), for both dependent variables, shows that most of the factors studied were statistically significant (*p* <0.05) in their linear terms;* p-*value for bixin (%) of X_2_-pH is 0.065, which is also >0.050.* p*-value for polyphenol of X_1_-treatment tine and X_3_X_4_ is >0.050, too.

**Figure 1:**
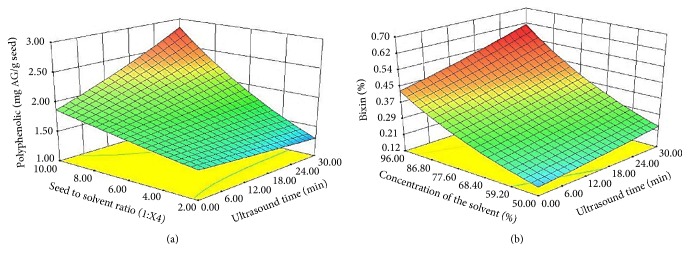
Response surface plots for the significant effects of seed-to-solvent ratio* vs.* treatment time and concentration of the solvent* vs*. treatment time for (a) polyphenol compounds and (b) bixin.

The statistical significance of the independent variables is attributed to the ionization states of the aliphatic molecules upon the modification of the pH and physical processes of mass transfer attributed to the effect of ultrasounds and solvent concentration. Bixin is a carotenoid having a highly conjugated structure and presents a carboxyl group. It is also highly soluble at basic pH. On the other hand, polyphenol compounds are slightly acidic molecules and their solubility is not significantly influenced by the medium pH. Therefore, the concentration of the extraction solvent (i.e., ethanol) was the variable of greater statistical significance for all compounds. The statistical significance of seed-to-solvent ratio was attributed to the saturation of the extraction medium. Further, the significance of the treatment time in the extraction process was related to the residence time of the cavitation bubbles in contact with the seeds, easing the release of the metabolites. During the first 15 minutes of ultrasound treatment, mass transfer occurred mainly by convection and corresponded to the solubility of the extracted solute, then diffusion phenomena took place involving the internal part of the seed particles where the extraction of the compounds is maximized [20, 21]. The response surfaces plots obtained from the polynomial models are shown in Figure 1. All the models were subjected to an optimization process. The polynomial equations for the response variables are described as follows:

(3) ln⁡bixin=−5.61+0.03∗X2+0.07∗X3+0.46X4−0.01∗X1−0.01∗X3∗X4+0.01∗X4∗X1

(4) ln⁡polyphenols=−0.17+0.01∗X3+0.03∗X4−0.01∗X1+0.01∗X4∗X1

The plots describe how the application of ultrasounds accelerates the extraction process of both type of bioactive compounds. Once both treatments were optimized, the estimated conditions for the UAE, along with their relative error are listed in Table 3. The absolute bias of the UAE process was high, and thus the experimental results obtained were greater than those predicted by the statistical analysis.

Results from the UAE were compared with a previously reported microwave-assisted extraction (MAE) conduced on the same seeds [12]. The MAE is based on the exposure of the extract to high-energy electromagnetic waves generating heating of medium and in turn, increasing the energy transfer rate. The reported extraction yield of polyphenol compounds (mg AG/g seed) and bixin (%) by MAE corresponds to 3.08 ± 0.01 and 0.58 ± 0.02, respectively. These yields are close to those obtained reported in this study for UAE (Table 3). A detailed analysis of the MAE showed a treatment time (5 min), solvent concentration (ethanol) (96%), and a solvent-to-seed ratio (5.95:1) as optimal. This means that a shorter treatment time is needed for MAE as compared to the UAE to get about the same yield [12]. Emine and collaborators obtained similar results when compared both extraction techniques for polyphenol compounds from nettle. In this case, they used a treatment of 10 min at 407 W retrieving gallic acid (1.125 mg/g db), caffeic acid (1.223 mg/g db), chlorogenic acid (4.798 mg/g db), p-coumaric acid (1.157 mg/g db), naringenin (5.582 mg/g db), and naringin (0665 mg/g db). These compounds were statistically comparable to those obtained by UAE conducted at 80% power and 30 min of treatment time (1.209, 1.289, 4.453, 1.100, 5.735, and 0.784 mg/g db, respectively). In annatto seeds MAE shows the same extraction efficiency in a shorter period of time [22]. The major difference between both techniques is focused on the diffusion resistance for the mass and heating transfer of bioactive compounds. Thus, the UAE generates microdomains and cavitations of the bubbles causing a greater temperature ramp, whereas the MAE changes the molecular rotation and ionic mobility of the medium generating heating in the whole system and not in form of microdomains.

### 3.2. Effect of the UAE on the Antimicrobial and Antioxidant Activities of the Extract

The antioxidant and antimicrobial properties of the extracts obtained at the optimal operational conditions for the UAE were compared with an extract obtained by conventional extraction and a previous study conducted by MAE.  Table 4 shows a larger antimicrobial and antioxidant activities for the UAE and MAE than that observed for the extract obtained by convectional extraction. This is explained by the higher content of polyphenol and bixin compounds present in both emerging extraction methods.

The extract of the seeds of annatto contains mainly bixin and polyphenol compounds which are responsible for the antioxidant and antimicrobial activities [2]. Particularly, bixin is a highly conjugated molecule and polyphenol compounds present hydroxyl groups in its structure having the ability to capture electrons, extinguishing the singlet oxygen, deactivating the excited triplet state of sensitizers which are usually associated with photosensitization and sweeping free radicals during their transition states. As a result, these compounds showed a large antioxidant activity. On the other hand, the antimicrobial activity is attributed mainly to the content of polyphenol compounds in the extract. These compounds have the ability to denature the proteins of microbial cell membranes and thus generate their inactivation or death, without being affected by the medium pH [2]. Consequently, the antimicrobial and antioxidant activities increased in the extracts obtained by UAE and MAE due to the greater amount of bioactive compounds in comparison to the extract obtained by leaching. Similar results were obtained by Zhang et al. (2010) and Yolmeh et al. (2014) when UAE was used for the extraction of astaxanthin and bixin respectively, showing an increased antioxidant capacity [3, 23].

### 3.3. Identification of Bixin and Polyphenol Compounds of the Extract Obtained by UAE under Optimal Conditions

**Figure 2:**
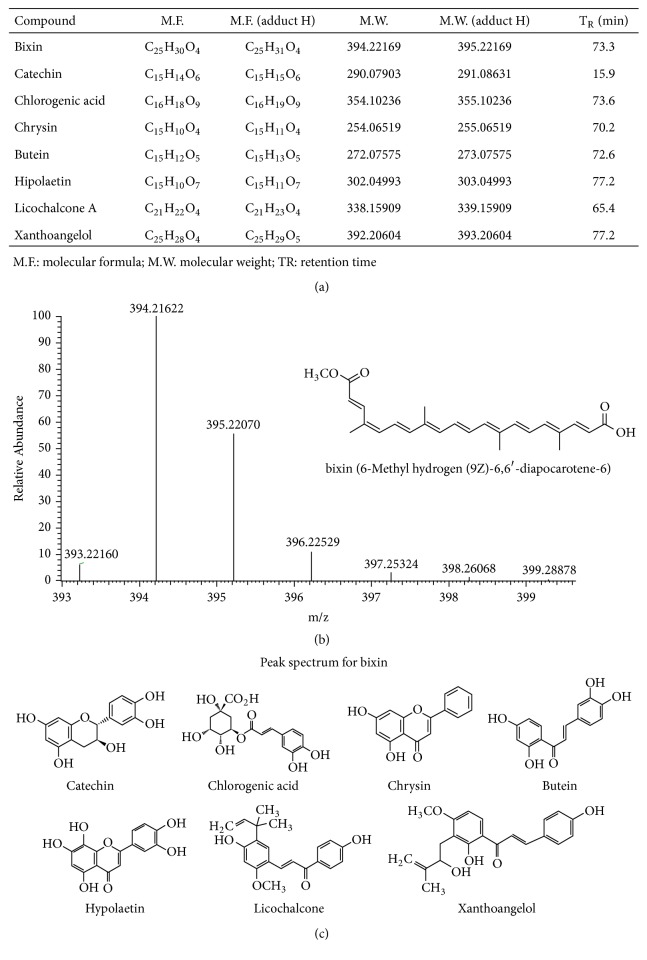
(a) Bioactive compounds identified in annatto seeds extract by LC/ESI-MS analysis. (b) Experimental MS2 spectrum of the ion at m/z 394.22169 with the predicted structures of the ion at m/z 395.22070 arising from bixin (6-Methyl hydrogen (9Z)-6,6′-diapocarotene-6). (c) Structures of polyphenol compounds identified from the annatto seeds extract.

The extract obtained by UAE was subjected to an LC/ESI-MS using an orbitrap as an MS analyzer for the identification bioactive compounds [2]. The monoisotopic mass was searched in a database of plant phenolic compounds and the results were listed in Figure 2. Seven polyphenol compounds were identified, including catechin (flavonoids-flavanols), chlorogenic acid or 5-caffeoylquinic acid (phenolic acids-hydroxycinnamic acids), chrysin (flavonoids-flavanols), butein (flavonoids-chalcone), hypolaetin (flavonoids-flavanols), licochalcone A (flavonoids-chalcone), xanthohumol (flavonoids-chalcone). Furthermore, compounds such as hypolaetin and chlorogenic acid or 5-Caffeoylquinic acid were previously identified by Campos et al. (2011) who designed a chromatographic method for the quantification of bixin and polyphenol compounds from the extract of annatto seeds. They reported exclusively bixin as the main carotenoid compound accounting for the 80% in the annatto seed extracts. Further, they also reported hypolaetin and caffeic acid derivatives as the main polyphenol compounds. However, they did not identify any other type of polyphenols in the extract [1, 10].

Despite the verified antioxidant activity of the compounds extracted from annatto seeds and identified by the orbitrap, some of them, particularly, catechin, chlorogenic acid, chrysin, and licochalcone A, present antimicrobial activity against gram (+), gram (-) bacteria, and fungi [24–27]. Further, Dzotam and collaborators in 2017 reported the antimicrobial activity of six Cameroonians food plants (*Psidium guajava, Persea americana, Citrus sinensis, Coula edulis, Mangifera indica*, and* Camellia sinensis*) against enteric multiresistant bacteria (*Providencia stuartii, Klebsiella pneumoniae, *and* Escherichia coli*). They declared that the antimicrobial activity is partly attributed to polyphenol compounds such as catechin. This compound either affects the membrane fluidity in the hydrophilic and hydrophobic regions of lipid bilayers of the microorganism, or inhibits the action of DNA polymerases [24]. Further, chlorogenic acid, chrysin, and licochalcone A have also been reported as polyphenol compounds with antimicrobial activity against* E. coli, M. Luteus,* and* S. aureus*. The latter is more susceptible due to differences of the proteins of the cell wall. For instance, the cell wall of E. coli contains of a thin layer of peptidoglycans and an external layer of lipoproteins, lipopolysaccharides, and phospholipids, whereas the cell wall of S. aureus includes a peptidoglycan layer with many pores. The porous structure of the cell wall of S. aureus makes it easy for interaction with chlorogenic acid or other antimicrobial compounds, inhibiting the synthesis of nucleic acids, the function of the cytoplasmic membrane, or the energetic metabolism [25–27].

## 4. Conclusions

The UAE technique accelerated the extraction process, reduced energy expenditure, and increased the yield of the bioactive compounds present in the annatto seeds. The UAE preserved the metabolites with a greater efficiency and thus favored their functional activities. In addition, the presence of bixin and seven polyphenol compounds such as catechin, chlorogenic acid, chrysin, butein, hypolaetin, licochalcone A, and xanthohumol found in the extract was responsible for their antimicrobial and antioxidant activities.

## Figures and Tables

**Table 1:** The experimental matrix for the UAE of bioactive compounds from annatto seeds.

Treatment number	UAE Treatment time (min)	pH	Solvent Concentration (%)	Seed-to solvent ratio (1:X_4_)	UAE					
					Polyphenol content (mg AG/g seed) n=3			Bixin content (%) n=3		
1	0.00	7.50	73.00	10.00	1.57	±	0.094	0.17	±	0.053
2	15.00	4.00	50.00	6.00	1.50	±	0.014	0.15	±	0.038
3	15.00	7.50	50.00	10.00	1.92	±	0.017	0.32	±	0.030
4	15.00	4.00	96.00	6.00	2.15	±	0.135	0.54	±	0.049
5	15.00	7.50	73.00	6.00	2.06	±	0.068	0.31	±	0.029
6	0.00	11.00	73.00	6.00	1.90	±	0.155	0.38	±	0.035
7	30.00	11.00	73.00	6.00	1.80	±	0.023	0.67	±	0.066
8	15.00	11.00	96.00	6.00	2.17	±	0.032	0.57	±	0.068
9	0.00	7.50	50.00	6.00	1.59	±	0.119	0.17	±	0.013
10	15.00	7.50	50.00	2.00	1.11	±	0.137	0.12	±	0.086
11	30.00	4.00	73.00	6.00	2.03	±	0.056	0.39	±	0.018
12	15.00	4.00	73.00	2.00	1.11	±	0.029	0.39	±	0.019
13	15.00	7.50	73.00	6.00	1.86	±	0.073	0.31	±	0.019
14	30.00	7.50	96.00	6.00	2.05	±	0.157	0.50	±	0.030
15	15.00	11.00	73.00	10.00	2.49	±	0.154	0.20	±	0.025
16	15.00	11.00	50.00	6.00	1.57	±	0.065	0.19	±	0.004
17	0.00	7.50	96.00	6.00	1.76	±	0.134	0.22	±	0.016
18	15.00	7.50	96.00	2.00	1.53	±	0.099	0.54	±	0.008
19	15.00	7.50	73.00	6.00	2.11	±	0.143	0.33	±	0.012
20	15.00	7.50	96.00	10.00	2.85	±	0.066	0.64	±	0.031
21	30.00	7.50	73.00	2.00	1.50	±	0.132	0.39	±	0.029
22	15.00	7.50	73.00	6.00	1.85	±	0.246	0.27	±	0.058
23	15.00	11.00	73.00	2.00	1.57	±	0.180	0.47	±	0.022
24	30.00	7.50	73.00	10.00	2.72	±	0.140	0.38	±	0.025
25	0.00	4.00	73.00	6.00	1.42	±	0.113	0.24	±	0.037
26	15.00	7.50	73.00	6.00	2.16	±	0.121	0.33	±	0.017
27	30.00	7.50	50.00	6.00	1.33	±	0.098	0.27	±	0.014
28	0.00	7.50	73.00	2.00	1.47	±	0.064	0.38	±	0.041
29	15.00	4.00	73.00	10.00	2.33	±	0.094	0.28	±	0.007
30	15.00	7.50	73.00	6.00	2.03	±	0.014	0.35	±	0.047

Values are expressed as mean ± standard deviation (n = 3). UAE: ultrasound-assisted extraction; AG: gallic acid.

**Table 2:** ANOVA table for the response variables for the UAE of bioactive compounds from annatto seeds.

*Variable*	*UAE*	
	Polyphenol (mg AG/g seed)	Bixin (%)
	p-value	p-value
Model	< 0.0001	< 0.0001
X_1_-Treatment time (min)	>0.050	0.006
X_2_-pH	>0.050	0.065
X_3_- Solvent concentration (%)	0.0001	< 0.0001
X_4_- Seed-to-solvent ratio (1:X_4_)	< 0.0001	0.003
X_3_X_4_	>0.050	< 0.0001
X_4_X_1_	0.036	0.023
Lack of Fit	0.058	0.057
r^2^	0,768	0,897
r^2^-adj	0,731	0,872

AG: gallic acid.

**Table 3:** Predicted local maximum in the optimization of the UAE applying a Box-Behnken experimental design.

Parameter	pH	Solvent concentration of the (%)	Seed-to-solvent ratio (1:X4)	Treatment time (min)	Polyphenol content (mg AG/g seed)	Bixin (%)
Predicted	7.5	95.98	7.82	30.00	2.71	0.43
Experimental	7.0	96.00	7.00	20.00	3.81	0.62
	Relative error				-1.10	-0.19
	Absolute error (%)				40.74	44.42

Values are expressed as mean ± standard deviation (n = 3). AG: gallic acid.

**Table 4:** Minimum inhibitory concentration (MIC) at different pHs against *B. cereus* and *S. aureus* and antioxidant activity of the extracts obtained by UAE and leaching.

Assay/extract		UAE	Leaching	MAE
*Bacillus cereus*	pH 11 (mg /L)	37 ^a^	48 ^b^	16 ^c^
	pH 7 (mg /L)	37 ^a^	48 ^b^	16 ^c^
	pH 4 (mg /L)	37 ^a^	48 ^b^	16 ^c^
*Staphylococcus aureus*	pH 11 (mg /L)	9 ^a^	48 ^b^	8 ^a^
	pH 7 (mg /L)	9 ^a^	48 ^b^	8 ^a^
	pH 4 (mg /L)	9 ^a^	48 ^b^	8^a^
Bixin	(%)	0.621^a^ ± 0.031	0.165^b^ ± 0.002	0.576 ^a^ ± 0.015
Polyphenols	mg AG/g seed	3.814^a^ ± 0.201	0.343^b^ ± 0.003	3.078 ^a^ ± 0.012
ABTS	*μ*M Trolox/L extract	1035.652^a^ ± 189.517	174.782^b^ ± 8.700	577.68 ^c^ ± 5
FRAP	*μ*M Trolox/L extract	424.700^a^ ± 7.000	127.033^b^ ± 2.517	316.37 ^c^ ± 10
DPPH	*μ*M Trolox/L extract	1161.524^a^ ± 28.938	811.048^b^ ± 5.774	1043.90 ^c^ ± 50

Values are expressed as mean ± standard deviation (n = 3). Different letters indicate statistically significant differences (p < 0.05).

## Data Availability

The data used to support the findings of this study are available from the corresponding author upon request.
